# Associations of obesity and novel lipid indicators in the risk of type 2 diabetes mellitus in Chinese elderly hypertensive patients

**DOI:** 10.3389/fendo.2025.1475323

**Published:** 2025-04-01

**Authors:** Xihui Jin, Yushuang Wei, YeMei Mo, Qiuling Zhang, Mingjie Xu, Xiaoyou Mai, Boteng Yan, Wenchao Xie, Shengzhu Huang, Mingli Li, Zengnan Mo

**Affiliations:** ^1^ Institute of Urology and Nephrology, First Affiliated Hospital of Guangxi Medical University, Guangxi Medical University, Nanning, Guangxi, China; ^2^ Center for Genomic and Personalized Medicine, Guangxi Key Laboratory for Genomic and Personalized Medicine, Guangxi Collaborative Innovation Center for Genomic and Personalized Medicine, Guangxi Medical University, Nanning, Guangxi, China; ^3^ School of Public Health, Guangxi Medical University, Nanning, Guangxi, China; ^4^ The First People’s Hospital of Yulin, Yulin, Guangxi, China

**Keywords:** obesity indices, novel lipid indicators, type 2 diabetes mellitus, elderly hypertensive patients, Chinese community

## Abstract

**Background:**

The associations of waist circumference (WC), body mass index (BMI), lipid accumulation product (LAP), Chinese visceral adiposity index (CVAI), and triglyceride-glucose Index (TyG) with the risk of type 2 diabetes mellitus (T2DM) remained uncertain in Chinese middle-aged and elderly hypertensive patients.

**Methods:**

A total of 1,965 hypertensive participants aged 45 years and elderly were included in the cross-sectional analysis, and 1,576 hypertensive participants without T2DM for the cohort analysis. In the cross-sectional study, binary logistic regression, restricted cubic splines (RCS), and receiver operating characteristic (ROC) were used to analyze the relationships between WC, BMI, LAP, CVAI, and TyG with and T2DM in hypertensive patients. In the cohort study, Kaplan-Meier survival analyses and Cox regression were further performed to determine the associations of these indicators with incident T2DM risk.

**Results:**

In the cohort study, there were 101 incident T2DM cases occurred during a median follow-up of 30 months, with an incident rate was 2.78 per 100 person-years. The cross-sectional study showed that the risk of T2DM increased significantly with higher quartiles of WC, BMI, LAP, CVAI, and TyG (all *P*_-trend_ < 0.001). In the cohort study, Cox regression model showed that WC (Q4 vs. Q1, HR = 3.30, 95% CI = 1.66-6.59), BMI (Q4 vs. Q1, HR = 2.38, 95% CI = 1.30-4.36), LAP (Q4 vs. Q1, HR = 5.15, 95% CI = 2.40-11.02), CVAI (Q4 vs. Q1, HR = 3.38, 95% CI = 1.76-6.50), and TyG (Q4 vs. Q1, HR = 5.76, 95% CI = 2.82-11.77) were associated with a higher risk of incident T2DM. RCS confirmed the positive dose-response relationships of WC, BMI, CVAI and TyG with T2DM in both study design, except for LAP in the cohort study. Additionally, ROC analysis revealed that TyG had the strongest area under the curve (AUC) of 0.70 (95% CI = 0.67-0.72) in the cross-sectional study, and the AUC of other indicators ranged from 0.55 to 0.57.

**Conclusion:**

Higher levels of WC, BMI, LAP, CVAI, and TyG are associated with a higher riskof developing incident T2DM in Chinese elderly hypertensive patients, and TyG might be the most effective predictive indicator.

## Background

With the aging of the global population and changing lifestyles, diabetes and hypertension have emerged as significant public health challenges worldwide (1). According to the International Diabetes Federation (IDF), there were approximately 537 million people aged 20-79 with diabetes worldwide in 2021, accounting for 10.5% of the global adult population, and the cases will rise to 783 million (12.2%) by 2045. China has the largest number of people with diabetes in the world, which is expected to reach 175 million (2). Data from the U.S. National Health and Nutrition Examination Survey (NHANES) showed that the prevalence of diabetes in people with high blood pressure is about two times than those with normal blood pressure. Relate study indicated that individuals with hypertension and diabetes comorbidity have much higher risks for cardiovascular diseases than those with single disease (3).

Obese patients face an increased risk of cardiovascular disease, which is widely recognized as a significant risk factor for type 2 diabetes mellitus (T2DM) (4). Although current clinical assessment includes routine evaluation indicators for identifying high-risk individuals for T2DM (5), there are limitations with traditional measures such as body mass index (BMI) and waist circumference (WC). BMI primarily assesses overall obesity does not reflect the distribution of body fat, while WC, although it can be used as a measure of abdominal obesity, has limitations in distinguishing subcutaneous adipose tissue from visceral adipose tissue, and may also exhibit biases across different body types, resulting in reduced accuracy (6).

In recent years, a number of novel lipid-related markers have emerged as predictors of T2DM risk. The lipid accumulation product (LAP), calculated from WC and triglyceride (TG) levels, are considered to be a more accurate indicator of metabolic risk associated with central obesity and excessive lipid accumulation. Higher LAP scores indicate greater degree accumulation of body fat (7). The Chinese Visceral Adiposity Index (CVAI), which combines age, BMI, WC, TG, and high-density lipoprotein cholesterol (HDL), is a surrogate biomarker for assessing visceral fat accumulation. Insulin resistance (IR) is recognized as a key pathological mechanism underlying a variety of diseases, including those affecting the heart, brain, and kidneys (8, 9). Additionally, the triglyceride-glucose (TyG) index, calculated from the product of TG and fasting plasma glucose (FPG), is regarded as an alternative marker for identifying insulin resistance and determining metabolic health status (10, 11). Several studies have indicated that a high TyG index is associated with an increased risk of T2DM in different ethnic groups (12, 13). Previous studies have shown that elevated BMI, WC, LAP, CVAI and TyG levels are associated with increased risk of T2DM (14, 15). However, most studies have been conducted in general population samples. In contrast, patients with hypertension have a higher incidence of diabetes (15, 16). Hypertension causes endothelial dysfunction, which promotes insulin resistance and diabetes onset (17, 18). Therefore, hypertension may be a predisposing factor for diabetes. However, research is limited as to whether these obesity and lipid-related markers are similarly predictive of T2DM in middle-aged and elderly hypertensive patients.

This study aims to comprehensively assess the predictive value of BMI, WC, LAP, CVAI, and TyG, in identifying individuals at risk for T2DM among hypertensive patients. The study will employ a two-pronged approach: first, a cross-sectional study to determine the relationship between these indicators and T2DM risk, identifying the most effective predictors. Secondly, a cohort study will evaluate the long-term predictive performance of these indicators on the incidence of diabetes. The goal is providing reliable and practical screening tools that can help healthcare professionals effectively identify and manage diabetes risk in hypertensive patients.

## Methods

### Study design and population

**Figure 1:**
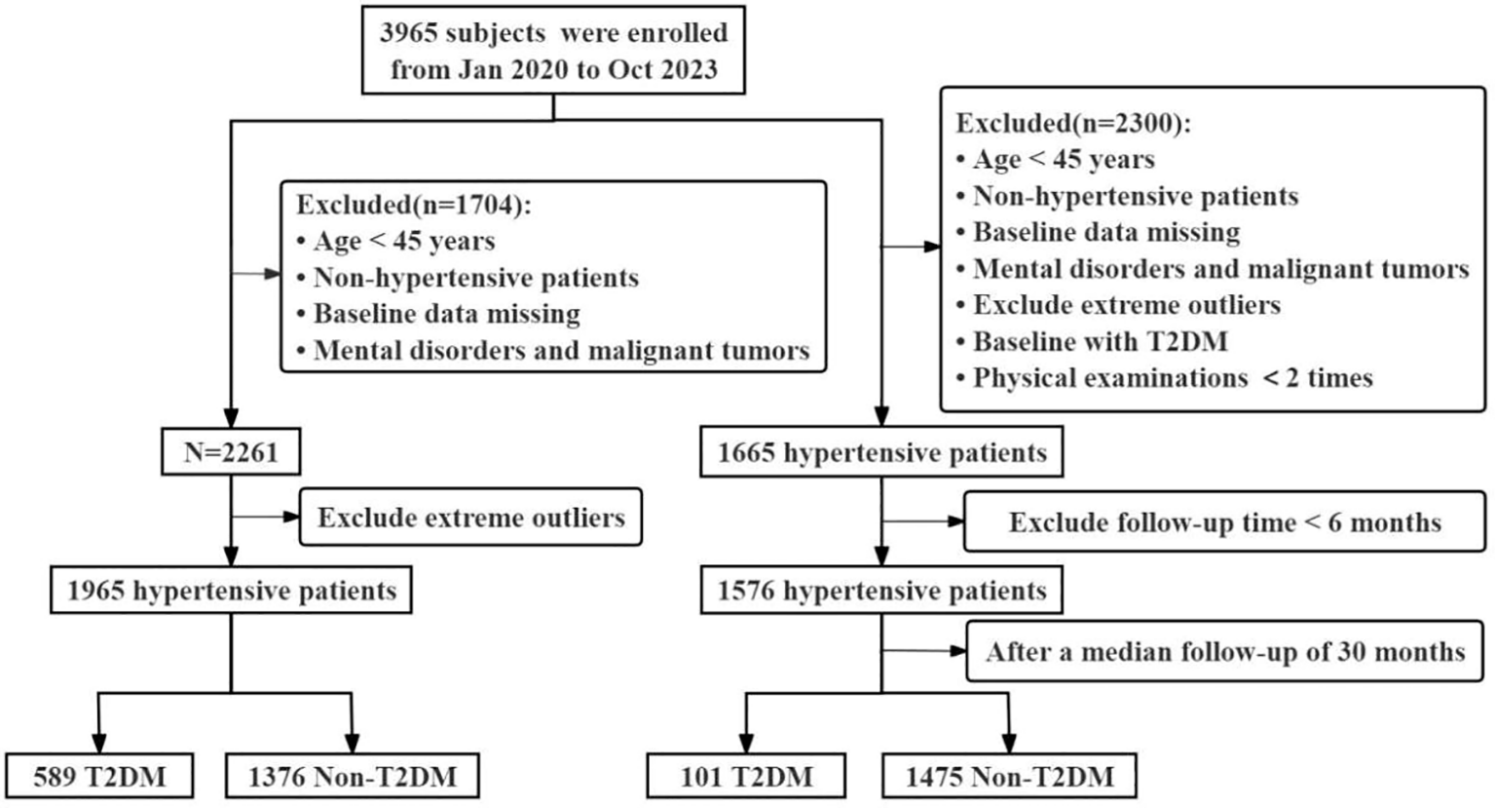
Flowchart of the study population.

We obtained data from the basic public health service (BPHS) management system of the Wuliqiao Community Health Service Center in Yulin city of Guangxi, and a total of 3,965 permanent residents recruited between January 2020 to October 2023 were included as the study subjects. The inclusion criteria for the subjects were as follows: (1) age ≥ 45 years; (2) hypertensive patients. Next, we excluded the subjects with the following criteria: (1) missing or extremely abnormal values in for age, blood pressure (BP), BMI, WC, TG, HDL-C, low-density lipoprotein cholesterol (LDL-C) and fasting plasma glucose (FPG); (2) suffered from mental illness or malignant tumors. Finally, a total of 1,965 participants (879 men and 1,086 women) were included in the cross-sectional study to investigate the relationships between WC, BMI, LAP, CVAI and TyG and T2DM risk in hypertensive patients with the most recent examination data. To further validate the associations between these indicators with incident T2DM, we further chose the hypertensive patients who were non-diabetic patients, having ≥ 2 times physical examinations and ≥ 6 months follow up period as the subjects in the cohort study, and then a total of 1,576 participants were included with a median follow-up period of 30 months. The flowchart for this study was shown in **Figure 1**. This study was received ethical approval by the Ethics and Human Subject Committee of Guangxi Medical University, China (No. 2022-0193), and the Ethics and Human Subject Committee of First Affiliated Hospital of Guangxi Medical University, China (No. 2023-K090-01).

### Data collection and definition

Questionnaire surveys, physical examinations and fasting blood samples were conducted by trained medical professionals from the community health service center. The questionnaire covered basic information, lifestyle, major health issues and health assessments, including gender (male, female), age (years), exercise (yes: exercising at least three times per week, with each session lasting at least 30 minutes, no: exercising less than this amount), smoking: (yes: current smoker, no: non-smoker or former smoker), alcohol drinking: (yes: consuming alcohol more than 12 times in the past 12 months, no: consuming alcohol less frequently).

### Anthropometry and biochemical measurements

Physical examinations included measurement of height, weight, WC and BP. For height and weight measurements, participants wore light clothing and no shoes. WC was measured at the midpoint between the lower rib margin and the iliac crest on the horizontal plane of the mid-axillary line, recorded at the end of expiration. Before BP measurement, participants were instructed to rest quietly for at least 5 minutes in a calm environment. They were seated with their upper arm placed on a flat surface, palm facing upward, ensuring the upper arm was at the same level as the heart. BP was measured twice at 30-second intervals using an Omron electronic blood pressure monitor and averaged for analysis. Hypertension was defined as systolic blood pressure (SBP) ≥140 mmHg and/or diastolic blood pressure (DBP) ≥90 mmHg. Patients remain categorized as hypertensive even if their blood pressure is lower than 140/90 mmHg while taking antihypertensive medication. Laboratory testing involved collecting fasting venous blood samples from study subjects to measure levels of FPG, TC, TG, LDL-C and HDL-C. T2DM was defined as fasting blood glucose≥7.0 mmol/L, or patients previously diagnosed with diabetes at township (community) level or higher hospitals. BMI, LAP, CVAI and TyG were calculated using the following formulas:


$$\text{BMI}=\text{weight}\left(\text{kg}\right)/{\text{height}}^{2}\left(\text{m}\right).$$

LAP(male)=[WC(cm)−65]×TG(mmol/L).

LAP(female)=[WC(cm)−58]×TG(mmol/L).

$$\text{CVAI}\left(\text{male}\right)=-267.93+0.68\times \text{age}\left(\text{y}\right)+0.03\times \text{BMI}\left(\text{kg}/{\text{m}}^{2}\right)+4.00\times \text{WC}\left(\text{cm}\right)+22.00\times \text{lg }\left[\text{TG }\left(\text{mmol}/\text{L}\right)\right]-16.32\times \text{HDL-C}\left(\text{mmol}/\text{L}\right).$$

$$\text{CVAI}\left(\text{female}\right)=-187.32+1.71\times \text{age}\left(\text{y}\right)+4.32\times \text{BMI}\left(\text{kg}/{\text{m}}^{2}\right)+1.12\times \text{WC}\left(\text{cm}\right)+39.76\times \text{lg}\left[\text{TG }\left(\text{mmol}/\text{L}\right)\right]-11.66\times \text{HDL-C}\left(\text{mmol}/\text{L}\right).$$

TyG=ln[TG(mg/dL)×FPG(mg/dL)/2].

### Statistical analysis

The Kolmogorov-Smirnov test was used to assess the normality of continuous variables. Normally distributed continuous variables are presented as mean and standard deviation (SD), while skewed continuous variables are presented as median with interquartile range (Q1, Q3). Categorical variables are presented as frequency and percentage. To assess group differences, the p-value was calculated by Pearson’s Chi-squared test or Wilcoxon rank sum test. In both the overall population and subgroup analyses, continuous variables such as WC, BMI, LAP, CVAI and TyG were categorized using quartiles. In the cross-sectional study, logistic regression models calculated odds ratios (OR) and 95% confidence intervals (95% CI) to determine the relationships between WC, BMI, LAP, CVAI, TyG and diabetes prevalence. Further use of restricted cubic splines (RCS) was employed to analyze the potential associations between various indicators and T2DM. Finally, The performance of these indicators in diagnosing T2DM was compared using receiver operating characteristic (ROC) curves, with predictive value evaluated based on the area under the ROC curve (AUC). In the cohort study, COX proportional hazards model analysis, RCS analysis, and Time ROC analysis were employed to further validate the findings obtained from cross-sectional research. Additionally, Kaplan-Meier (KM) curve analysis was used to assess the risk of developing T2DM over time among quartiles of these indicators. All analyses were performed using R statistical software version 4.2.2 and SPSS version 26.0 for Windows. Statistical significance was defined as *P* < 0.05.

## Results

### Baseline characteristics of the study participants

Of 1,965 hypertensive participants in the cross-sectional study, the mean age was 70.26 ± 9.46 years old, and 589 were T2DM (29.97%). Most of them were female (55.27%), non-smokers (89.67%), non-drinker (89.57%), and having exercise every day (88.40%). Of 1576 hypertensive participants without T2DM in the cohort study, there were incident T2DM cases occurred during of 3644.08 person-years of follow-up (median 30 months), with the incident rate of T2DM was 2.78 per 100 person-years. The baseline characteristics of the follow-up subjects were similar with those in the cross-sectional study. Besides, statistically significant differences were observed in DBP, WC, FPG, TC, TG, HDL-C, BMI, LAP, CVAI and TyG between T2DM and non-T2DM groups in both the cross-sectional study and the cohort study (*P* < 0.05). The baseline features of the study participants were shown in **Table 1**.

**Table 1:** Baseline characteristics of the subjects in the cross-sectional study and cohort study.

Cross-sectional study					Cohort study			
Variables	Total (N=1,965)	Non-T2DM (N=1,376)	T2DM (N=589)	*P* value	Total (N=1,576)	Non-T2DM (N=1,475)	T2DM (N=101)	*P* value
Gender,n (%)				0.518				0.278
male	879 (44.73%)	609 (44.26%)	270 (45.84%)		683 (43.34%)	634 (42.98%)	49 (48.51%)	
female	1,086 (55.27%)	767 (55.74%)	319 (54.16%)		893 (56.66%)	841 (57.02%)	52 (51.49%)	
Age (yeas)	70.26 ± 9.46	70.13 ± 9.53	70.56 ± 9.30	0.671	68.67 ± 9.29	68.67 ± 9.36	68.53 ± 8.29	0.514
SBP (mmHg)	138.98 ± 14.29	139.36 ± 14.41	138.09 ± 13.99	0.054	138.58 ± 12.05	138.74 ± 12.12	136.28 ± 10.76	0.025
DBP (mmHg)	82.86 ± 8.40	83.27 ± 8.43	81.89 ± 8.27	<0.001	82.95 ± 7.56	83.06 ± 7.65	81.34 ± 6.00	0.032
WC (cm)	87.67 ± 8.18	87.23 ± 8.25	88.70 ± 7.93	<0.001	87.61 ± 8.84	87.38 ± 8.85	91.03 ± 7.99	<0.001
FPG (mmol/L)	4.93 (4.33,5.84)	4.61 (4.16,5.19)	6.3 (5.4,7.63)	<0.001	4.86 ± 0.76	4.81 ± 0.73	5.61 ± 0.82	<0.001
TC (mmol/L)	5.03 ± 1.12	5.07 ± 1.06	4.94 ± 1.24	0.007	5.02 ± 1.03	5.03 ± 1.04	4.80 ± 0.90	0.041
TG (mmol/L)	1.38 (1.02,1.93)	1.34 (1.01,1.85)	1.49 (1.05,2.11)	<0.001	1.35 (0.99,1.82)	1.32 (0.98,1.80)	1.63 (1.25,2.20)	<0.001
LDL-C (mmol/L)	3.05 ± 0.97	3.09 ± 0.94	2.96 ± 1.05	0.004	3.13 ± 0.93	3.14 ± 0.93	3.01 ± 0.90	0.188
HDL-C (mmol/L)	1.25 (1.05,1.51)	1.29 (1.07,1.54)	1.18 (1.02,1.43)	<0.001	1.29 (1.08,1.54)	1.30 (1.09,1.55)	1.12 (0.99,1.38)	<0.001
BMI (kg/m^2^)	24.13 ± 2.90	23.97 ± 2.94	24.50 ± 2.78	<0.001	24.12 ± 3.01	24.04 ± 3.00	25.28 ± 3.04	<0.001
LAP	36.48 (24.2,55.2)	35.2 (23.22,52.85)	38.42 (26.88,61.42)	<0.001	35.37 (22.96,53.19)	34.40 (22.55,52.03)	45.87 (32.24,67.65)	<0.001
CVAI	122.65 ± 30.99	120.26 ± 31.03	128.24 ± 30.19	<0.001	119.29 ± 32.65	118.26 ± 32.47	134.32 ± 31.64	<0.001
TyG	8.70 ± 0.63	8.56 ± 0.54	9.02 ± 0.69	<0.001	8.58 ± 0.53	8.56 ± 0.53	8.91 ± 0.53	<0.001
Exercise,n (%)				0.683				0.395
Yes	1,737 (88.40%)	1,219 (88.59%)	518 (87.95%)		1,447 (91.81%)	1,352 (91.66%)	95 (94.06%)	
No	228 (11.60%)	157 (11.41%)	71 (12.05%)		129 (8.19%)	123 (8.34%)	6 (5.94%)	
Smoking,n (%)				0.111				0.221
No	1,762 (89.67%)	1,224 (88.95%)	538 (91.34%)		1,480 (93.91%)	1,388 (94.10%)	92 (91.09%)	
Yes	203 (10.33%)	152 (11.05%)	51 (8.66%)		96 (6.09%)	87 (5.90%)	9 (8.91%)	
Alcohol drinking,n (%)				0.092				0.409
No	1,760 (89.57%)	1,222 (88.81%)	538 (91.34%)		1,481 (93.97%)	1,388 (94.10%)	93 (92.08%)	
Yes	205 (10.43%)	154 (11.19%)	51 (8.66%)		95 (6.03%)	87 (5.90%)	8 (7.92%)	

SBP, systolic blood pressure; DBP, diastolic blood pressure; WC, waist circumference; FPG, Fasting plasma glucose; TC, total cholesterol; TG, total triglyceride; LDL-C, low-density lipoprotein cholesterol; HDL-C, high-density lipoprotein cholesterol; BMI, body mass index; LAP, lipid accumulation product; CVAI, Chinese visceral adiposity index; TyG, triglyceride-glucose.

Mean ± SD for continuous variables with normal distribution, otherwise median (quartiles). The *P* value was calculated by Pearson’s Chi-squared test or Wilcoxon rank sum test.

### Associations of obesity and novel lipid indicators with T2DM in the cross-sectional study

**Figure 2:**
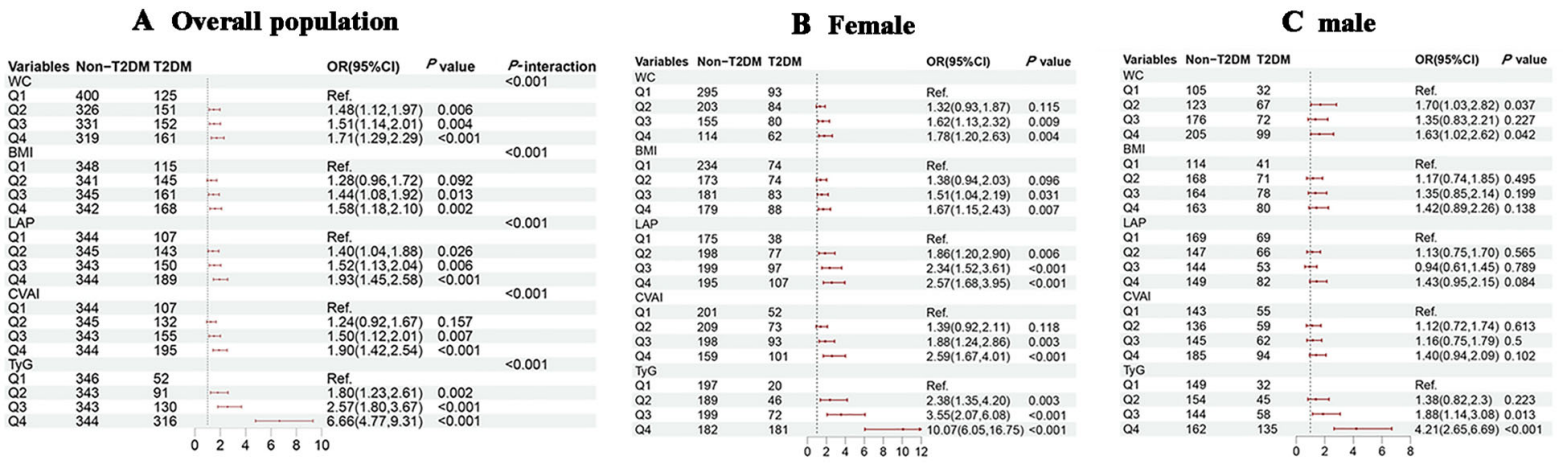
Associations of obesity and novel lipid indicators with T2DM in hypertensive patients in the cross-sectional study. **(A–C)** represent the overall population, females, and males in subgroup stratification, respectively. 95%CI, 95% confidence interval; OR, odds ratio; WC waist circumference, BMI body mass index, LAP lipid accumulation product, CVAI Chinese visceral adiposity index, TyG triglyceride-glucose. Adjustment for sex, age, systolic blood pressure, low-density lipoprotein cholesterol, exercise, smoking and alcohol drinking.

Univariate logistic regression model showed that the risk of T2DM increased significantly with higher quartiles of WC, BMI, LAP, CVAI, and TyG (all *P_-_*_trend_ < 0.001). After adjusting for confounders, the highest quartile of WC (OR = 1.71, 95% CI = 1.29-2.29), BMI (OR = 1.58, 95% CI = 1.18-2.10), LAP (OR = 1.93, 95% CI = 1.45-2.58), CVAI (OR = 1.90, 95% CI = 1.42-2.54), and TyG (OR = 6.66, 95% CI = 4.77-9.31) had the highest risk for T2DM when compared to the lowest quartile group. In subgroup analysis, positive associations of each study indicator with T2DM risk were consistently observed in female hypertensive patients (all *P* < 0.05), while positive associations between WC (Q4 vs. Q1, OR = 1.63, 95% CI = 1.02-2.62), TyG (Q4 vs. Q1, OR = 4.21, 95% CI = 2.65-6.69) with T2DM risk were found in male hypertensive patients (all *P* < 0.05) (**Figure 2**). In the aged 45-69 year group, only TyG demonstrated a significant increasing trend in the T2DM risk across quartiles, with 5.55-fold higher risk (95% CI = 3.32-9.90) in the fourth quartile compared to the first quartile. However, among patients aged 70 years and above, all five indicators exhibited a gradual increase in T2DM risk with increasing quartiles (**Supplementary Figure S1**). Gender and age significantly interacted with the association between WC, BMI, CVAI, and the TyG index and the risk of T2DM(all *P*_-interaction_ < 0.05).

### Associations of obesity and novel lipid indicators with T2DM in the cohort study

**Figure 3:**
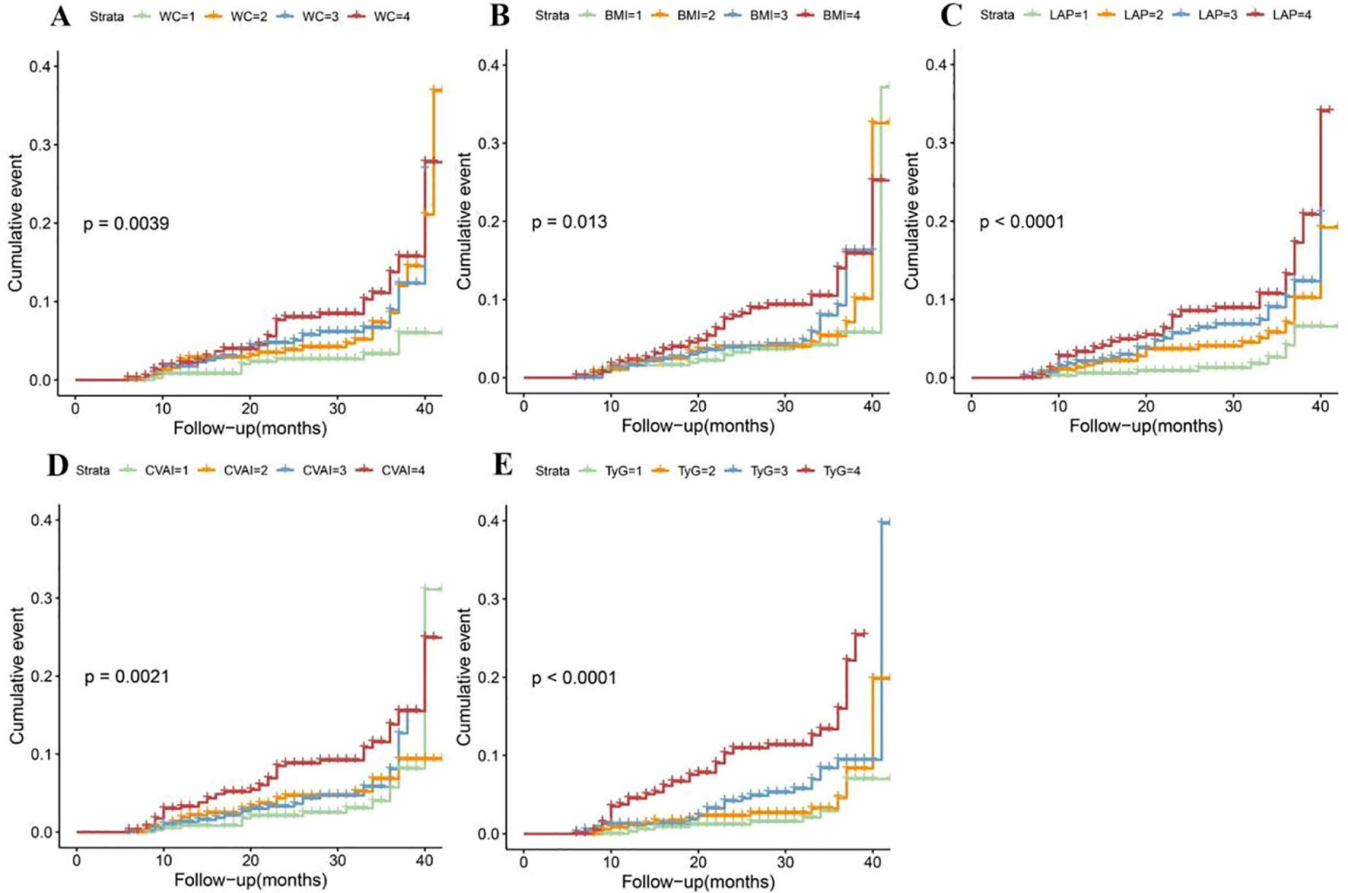
Kaplan-Meier cumulative event of T2DM in hypertensive patients according to the quartiles of obesity and novel lipid-related indicators. **(A)** waist circumference (WC); **(B)** body mass index (BMI); **(C)** lipid accumulation product (LAP); **(D)** Chinese visceral adiposity index (CVAI) and **(E)** triglyceride-glucose Index (TyG).

**Figure 4:**
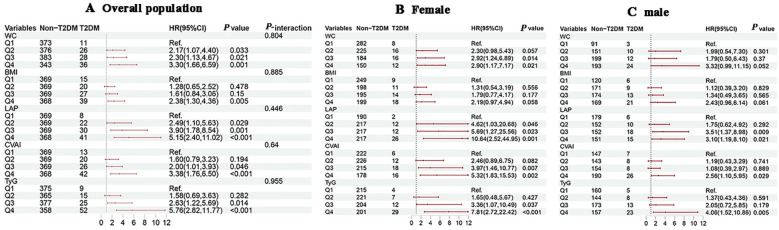
Associations of obesity and novel lipid indicators with the risk of T2DM in hypertensive patients in the cohort study. **(A–C)** represent the overall population, females, and males in subgroup stratification, respectively. 95%CI, 95% confidence interval; HR, hazard ratio; WC, waist circumference, BMI, body mass index, LAP, lipid accumulation product, CVAI, Chinese visceral adiposity index, TyG, triglyceride-glucose. Adjustment for sex, age, systolic blood pressure, low-density lipoprotein cholesterol, exercise, smoking and alcohol drinking.

KM survival analysis indicated that the cumulative incidence of T2DM increased significantly with higher quartiles of WC, BMI, CVAI and TyG (all log-rank test *P* < 0.05) (**Figure 3**). Multivariable Cox proportional hazards model analysis found that WC (Q4 vs. Q1, HR = 3.30, 95% CI = 1.66-6.59), BMI (Q4 vs. Q1, HR = 2.38, 95% CI = 1.30-4.36), LAP (Q4 vs. Q1, HR = 5.15, 95% CI = 2.40-11.02), CVAI (Q4 vs. Q1, HR = 3.38, 95% CI = 1.76-6.50), and TyG (Q4 vs. Q1, HR = 5.76, 95% CI = 2.82-11.77) were associated with a higher risk of incident T2DM (**Figure 4A**).

Subgroup analysis by gender showed that with increasing quartiles of each indicator, females consistently exhibited a higher risk of T2DM development with increasing quartiles of each indicator compared to males (**Figures 4B, C**). In the age-subgroup analysis, there was no substantial difference in the independent risk of developing T2DM across the age groups, but the risk was significantly higher in the fourth quartile (**Supplementary Figure S2**). The results indicated that age was significantly interacted the association between WC, TyG, and T2DM risk (*P*_-interaction_ < 0.05), and no significant interactions were observed between gender, age, and the BMI, CVAI, or LAP index.

### Dose-response relationships of obesity and novel lipid indicators with T2DM in hypertensive patients

**Figure 5:**
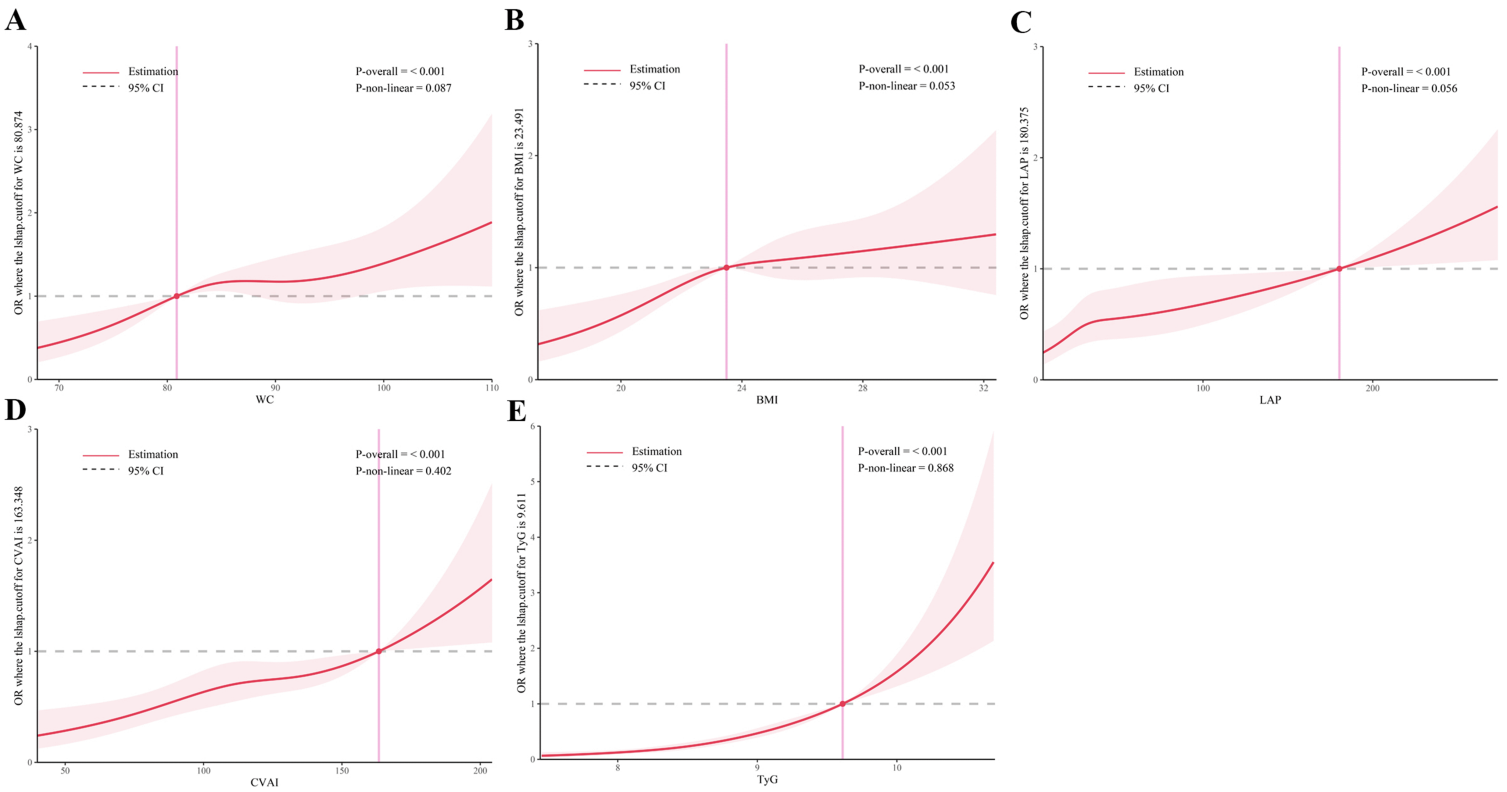
Restricted cubic spline analysis of obesity and novel lipid indicators with T2DM in hypertensive patients in the cross-sectional study. **(A)** waist circumference (WC), **(B)** body mass index (BMI), **(C)** lipid accumulation product (LAP), **(D)** Chinese visceral adiposity index (CVAI), and **(E)** triglyceride-glucose Index (TyG). Potential nonlinear relationships were examined using restricted cubic splines, with odd ratios (ORs) based on logistic regression models. The ORs was adjusted for sex, age, systolic blood pressure, low-density lipoprotein cholesterol, exercise, smoking and alcohol drinking.

**Figure 6:**
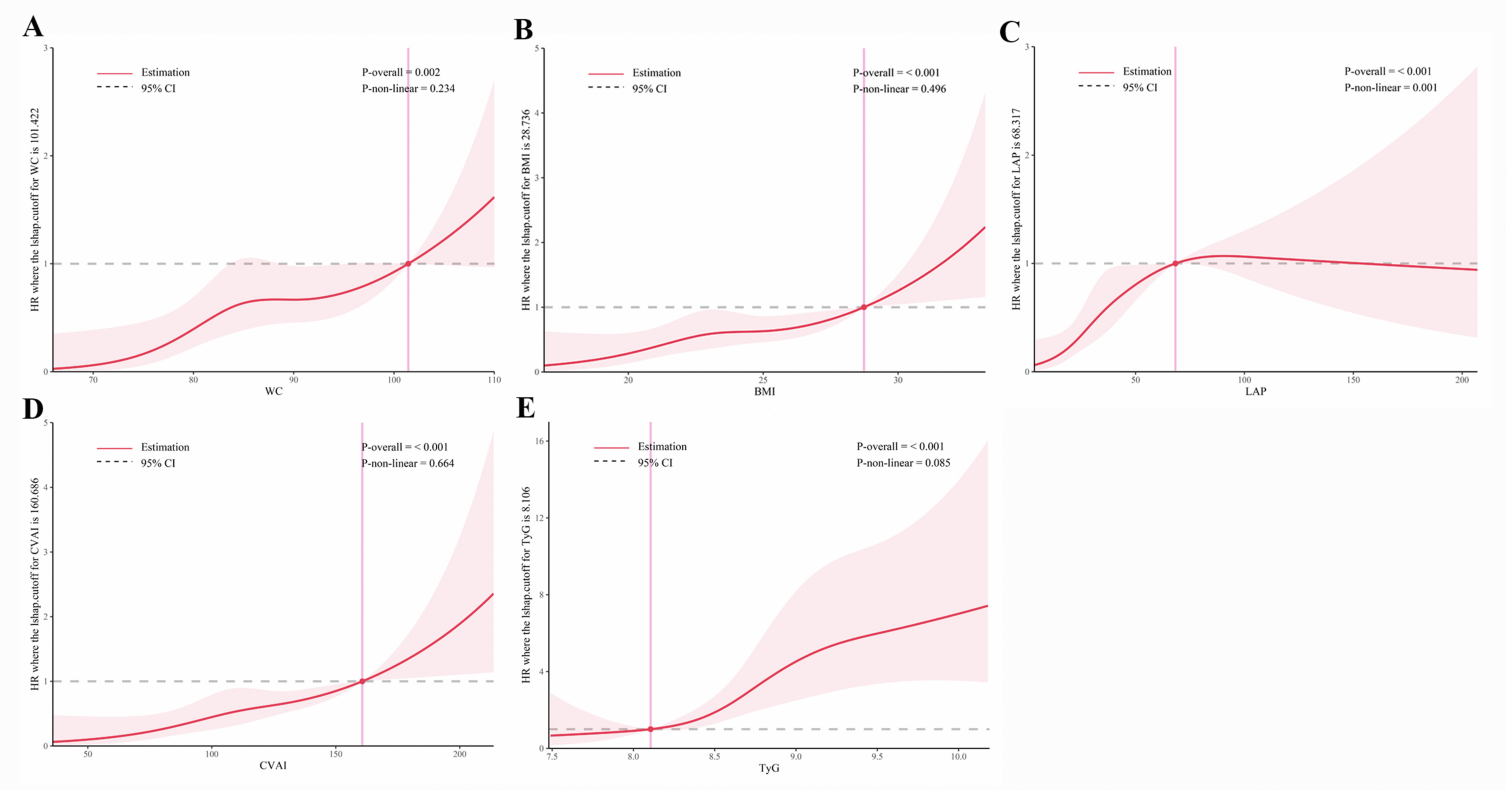
Restricted cubic spline analysis of obesity and novel lipid indicators with incident T2DM in hypertensive patients in the cohort study. **(A)** waist circumference (WC), **(B)** body mass index (BMI), **(C)** lipid accumulation product (LAP), **(D)** Chinese visceral adiposity index (CVAI), and **(E)** triglyceride-glucose Index (TyG). Potential nonlinear relationships were examined using restricted cubic splines, with hazard ratios (HRs) based on cox regression models. The HRs was adjusted for sex, age, systolic blood pressure, low-density lipoprotein cholesterol, exercise, smoking and alcohol drinking.

As shown in **Figures 5** and **6**, RCS analysis indicated linear dose-response relationships between WC, BMI, CVAI, and TyG with T2DM risk in both the cross-sectional and cohort studies (all *P*_overall_ < 0.001, *P _n_*_on-linear_ > 0.05). Of note, LAP demonstrated a positive linear association in the cross-sectional study (*P*_overall_ < 0.001, *P*_non-linear_ = 0.056), but a non-linear association in the cohort study (*P*_overall_ < 0.001, *P*_non-linear_ = 0.001).

### The predictive values of obesity and novel lipid indicators for T2DM

**Figure 7:**
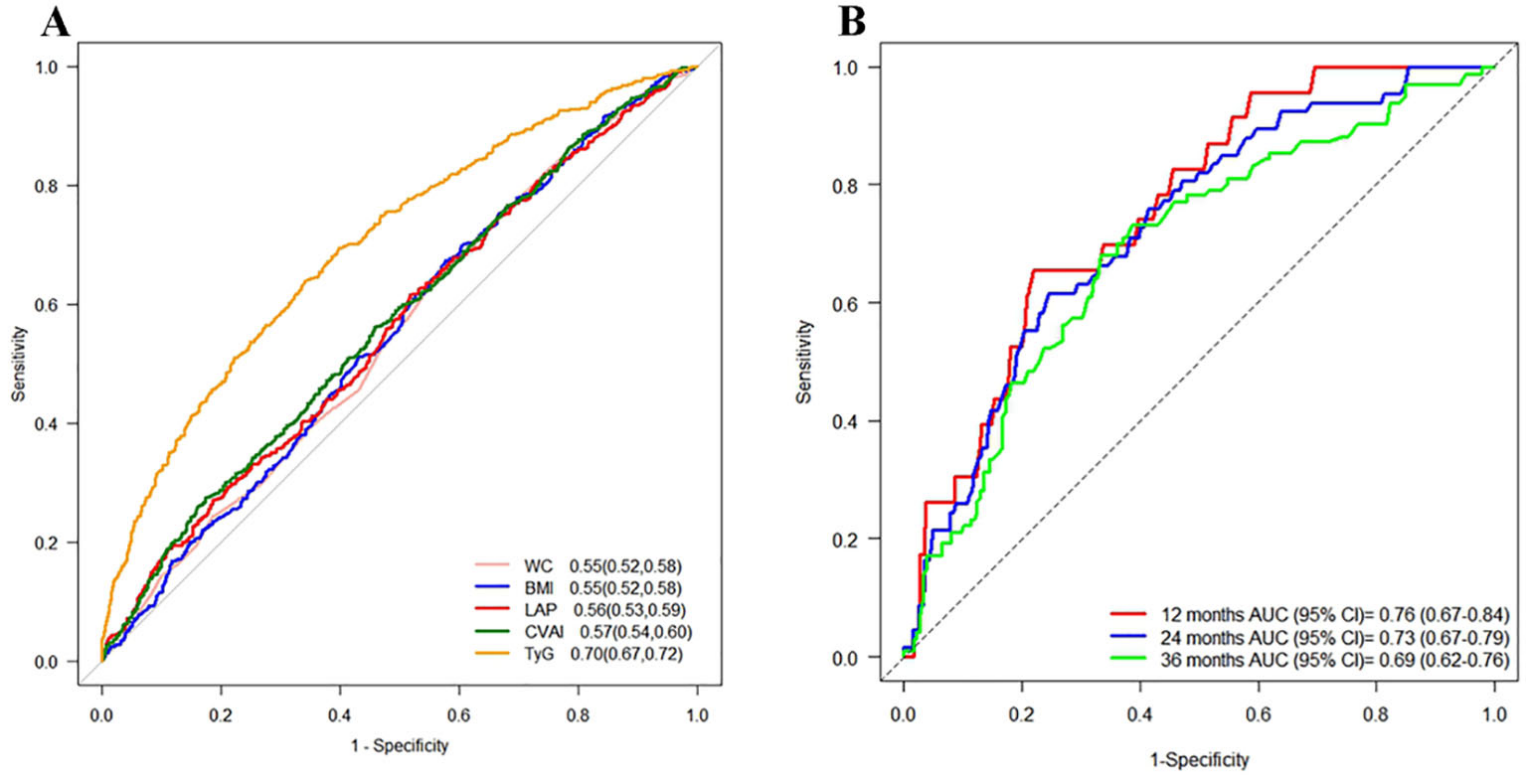
ROC curves for each index as predictors of T2DM. Footnotes are areas under the curve and 95% confidence intervals. **(A)** Cross-sectional study, **(B)** Time ROC curves of the TyG index at different time points in the cohort study.

In the cross-sectional study, TyG exhibited the highest AUC (0.70, 95% CI: 0.67-0.72) for T2DM, while other indicators showed moderate AUC values ranging from 0.55 to 0.57. In the cohort study, TyG still demonstrated the highest predictive ability for T2DM in the Time ROC analysis, with an AUC of 0.76 (95% CI: 0.67-0.84) at 12 months, but its predictive performance gradually declined over time (**Figure 7**). The time-dependent AUC values of other indicators ranged from 0.54 to 0.71 between 12 months to 36 months (**Supplementary Figure S3**). These findings suggested that TyG has the strongest predictive ability for T2DM onset compared to other indicators in both study settings.

## Discussion

This study investigated the relationships between five indices (WC, BMI, LAP, CVAI, and TyG) and T2DM in middle-aged and elderly hypertensive patients, and further assessed their predictive abilities for T2DM risk. All five indices were found to be independent risk factors for T2DM, with WC, BMI, CVAI, and TyG demonstrating a positive correlation with diabetes risk, indicating higher levels in patients with T2DM. Moreover, higher levels of these indices were associated with greater risk of developing T2DM. Additionally, ROC analysis revealed that TyG exhibited the strongest predictive ability for T2DM onset.

While traditional measures like WC and BMI are commonly used to assess obesity and T2DM risk (19, 20), emerging evidence suggests that novel lipid-related indices such as LAP, CVAI and TyG may more precisely reflect metabolic risk. Although our study similarly confirmed that WC and BMI were positively associated with T2DM risk in the general adult population, some studies have suggested that the association of LAP and CVAI with T2DM risk may be stronger than that of the traditional obesity indicators (WC, BMI). This is attributed to the inability of BMI to differentiate between fat mass and lean body mass and reflect fat distribution (21). Although WC can reflect abdominal fat distribution, it has limitations in distinguishing between subcutaneous fat tissue and visceral fat tissue, and it may cause significant biases in individuals with varying muscle mass. Growing evidence suggests that visceral fat tissue plays a more important role in insulin resistance and the development of diabetes than subcutaneous fat. Visceral fat produces more free fatty acids, inflammatory cytokines, and adipokines than subcutaneous fat, thereby increasing the risk of insulin resistance and diabetes (22–24). Adiponectin plays a critical role in metabolic signaling by exerting anti-inflammatory and insulin-sensitizing effects, primarily through the activation of AMP-activated protein kinase (AMPK) and peroxisome proliferator-activated receptor-α (PPAR-α) pathways (25). However, hypoadiponectinemia is frequently observed in hypertension associated with visceral obesity, contributing to endothelial dysfunction, systemic inflammation, and the exacerbation of insulin resistance and heart failure progression (26). These pathophysiological mechanisms may, at least in part, explain the increased risk of type 2 diabetes mellitus (T2DM) in hypertensive patients with elevated visceral adiposity indexes. This emphasizes the need for further research on these new lipid-related markers to better understand their predictive ability and value for clinical application in assessing T2DM risk.

LAP, a reliable indicator of visceral fat that combines WC and TG levels (27), has been shown to be a strong predictor of metabolic syndrome and pre-existing T2DM risk, with superior predictive ability to WC and BMI (28–30). A recent cohort study involving 15,717 Chinese participants showed that elevated LAP was positively associated with an increased risk of developing T2DM, suggesting that LAP may be a potential predictor of T2DM (31). Similar results have been found in Japanese and Korean populations (32, 33). Our study, using RCS analysis, revealed a strong association between LAP and T2DM risk, linear relationship in the cross-sectional study and a non-linear relationship in the cohort study, which may be due to the population selection. The results of our cohort study are consistent with a recent cross-sectional study involving 5,321 hypertensive patients in Guangzhou, China (34).

CVAI is a reliable lipid index that has been shown to be a reliable lipid predictor of diabetes risk events among Chinese individuals (35, 36), and it has a superior predictive ability for T2DM and prediabetes in Chinese adults compared to WC and BMI (37). Although our study did not find that LAP and CVAI were significantly better than WC and BMI in predicting diabetes risk, it is consistent with the results of a 2020 survey of 9,519 participants aged 60 and above in Zhongshan City, China, which found a linear correlation between CVAI levels and T2DM risk (38).

These findings, in combination with previous studies, suggest that elevated levels of LAP and CVAI are associated with an increased risk of T2DM in hypertensive patients and these associations present in all stratified variables, suggesting that LAP and CVAI may be early markers of T2DM risk in hypertensive patients. Further studies are needed to better understand their predictive ability and their value for clinical application in assessing T2DM risk.

The TyG index, calculated from FPG and TG levels (39), serves as a valuable surrogate marker for insulin resistance and has been consistently shown to predict cardiovascular events and diabetes development (40). Numerous studies have independently demonstrated its ability to predict new cardiovascular events. Investigations conducted in Asia and Europe consistently shown a strong correlation between TyG and the incidence of diabetes (32, 39, 41). For instance, Xiaoyun Zhang et al. examined 9,488 Chinese individuals aged 45 and above, identifying higher values of 13 obesity and lipid-related indicators metrics were associated with increased risk of T2DM (42). Among these indicators, TyG proved to be the strongest predictor of T2DM. Moreover, other studies have consistently shown a significant increase in diabetes risk with higher TyG index values (43–45). These findings are consistent with ours. The significant association between TyG index and T2DM is consistent with findings of a cross-sectional study involving 5,321 hypertensive patients in Guangzhou (34). However, their study did not identify a positive linear association, which possibly due to population selection and age demographics differences. A subgroup analysis of 201,298 Chinese individuals by Li et al. (46). revealed a positive association between TyG index and diabetes risk in subjects with SBP ≥140 mmHg and DBP ≥90 mmHg. Nonetheless, preventive measures for T2DM are lacking in middle-aged and older hypertensive populations. These findings emphasize the potential of the TyG index as a critical indicator of T2DM risk in these demographic groups.

In the cross-sectional study, gender and age significantly interacted with the associations between WC, BMI, CVAI, and TyG index and the risk of T2DM in middle-aged and elderly hypertensive individuals (all interaction *P*_-values_ < 0.05). However, in the cohort study, only age demonstrated a significant interaction with the TyG index regarding the risk of T2DM. This discrepancy may be due to differences in study design and time scale. The cross-sectional study captures associations between variables at a single point in time, whereas the cohort study examines the long-term effects of these variables on disease progression over time. In the cohort study of middle-aged and elderly individuals, changes in physical function, lifestyle, disease progression, or treatment over time may have interfered or diminished the observed interactions. Notably, while these interactions did not reach statistical significance in the cohort study, existing literature suggested that gender and age can influence the relationships between metabolic indicators and T2DM risk in certain contexts. For example, gender differences may affect metabolic indicators through mechanisms such as hormone levels and fat distribution (47, 48). Sex hormones, particularly estrogen and testosterone, play a pivotal role in regulating adipose tissue metabolism and insulin sensitivity. In females, estrogen is associated with reduced visceral fat accumulation and increased adiponectin secretion, which may partly explain the stronger associations between obesity indices (e.g., BMI and CVAI) and T2DM risk observed in women in our cohort (49). Conversely, lower testosterone levels in males are linked to increased visceral adiposity and insulin resistance, which may contribute to the higher baseline T2DM risk observed in hypertensive men (50). Future prospective studies are warranted to investigate whether sex hormone imbalances mediate the gender-specific relationships between lipid-related indices and diabetes risk. whereas age-related factors may be associated with changes in sarcopenia and insulin sensitivity (51–53). Therefore, the absence of significant interactions in our cohort study may be attributed to the complex interplay of other factors over the course of long-term follow-up.

The strengths of this study include its combined use of cross-sectional and cohort study methods. Based on middle-aged and elderly hypertensive patients in China, it provided causal validation of the relationships between WC, BMI, LAP, CVAI, TyG indices, and the risk of T2DM. By comparing traditional obesity indicators with novel lipid-related indicators, the study enhances the reliability of its findings and emphasizes the importance of exploring these newer measures in assessing metabolic risk. The study’s utilization of simple and routine examination items makes it practical and adaptable for large-scale T2DM screening in community settings, thus contributing to early detection and prevention strategies.

Our study also has some limitations. Firstly, this study did not observe significant associations in the 45-69 age group. This may be attributed to our study population being predominantly composed of elderly patients with hypertension (mean age >68 years), resulting in an insufficient sample size in the 45-69 age subgroup. However, statistical power analysis indicated that the TyG index exhibited high statistical power, with a value of 1.0 in both the cross-sectional and cohort studies. The statistical power of CVAI in the cohort study was 0.98, while the statistical power in the cross-sectional studywas <0.8. The statistical power values of WC, BMI, and LAP in both studies were < 0.8, which may explain the failure to detect potential associations. Future research should address these limitations by incorporating larger sample sizes from this population and conducting multicenter studies to further explore these relationships. Secondly, the study’s focus on middle-aged and elderly hypertensive patients from a specific community may limit generalizability, as differences in dietary intake and hypertension medications usage could influence blood pressure and glucose levels. Further research should explore the impact of these factors on the study’s findings. Finally, reliance on self-reported T2DM diagnosis or fasting venous blood glucose, without oral glucose tolerance tests or glycated hemoglobin testing, may underestimate the strength of these associations observed.

## Conclusions

In summary, higher levels of WC, BMI, LAP, CVAI, and TyG are significantly associated with higher odds of developing incident T2DM in Chinese elderly hypertensive patients, and TyG presents the strongest predictive ability for T2DM onset.

## Data Availability

The original contributions presented in the study are included in the article/**Supplementary Material**. Further inquiries can be directed to the corresponding authors.

## Supplementary material

Supplementary Figure 1 Age subgroup analysis in the cross-sectional study. **(A)** corresponds to Overall population, **(B)** corresponds to ages 45-69, and **(C)** corresponds to ages 70 and above. 95%CI, 95% confidence interval; OR, odd ratio; WC waist circumference, BMI body mass index, LAP lipid accumulation product, CVAI Chinese visceral adiposity index, TyG triglyceride-glucose. Adjustment for sex, age, systolic blood pressure, low-density lipoprotein cholesterol, exercise, smoking and alcohol drinking.

Supplementary Figure 2 Age subgroup analysis in the cohort study. **(A)** corresponds to Overall population, **(B)** corresponds to ages 45-69, and **(C)** corresponds to ages 70 and above. 95%CI, 95% confidence interval; HR, hazard ratio; WC waist circumference, BMI body mass index, LAP lipid accumulation product, CVAI Chinese visceral adiposity index, TyG triglyceride-glucose. Adjustment for sex, age, systolic blood pressure, low-density lipoprotein cholesterol, exercise, smoking and alcohol drinking.

Supplementary Figure 3 Time ROC curves of each index at different time points in the cohort study. **(A–D)** represent waist circumference (WC), body mass index (BMI), lipid accumulation product (LAP), and Chinese visceral adiposity index (CVAI), respectively.
