# Supplementary figures and images for: Associations of obesity and novel lipid indicators in the risk of type 2 diabetes mellitus in Chinese elderly hypertensive patients
Source: Front Endocrinol (Lausanne). 2025 Apr 1;16:1475323. doi: 10.3389/fendo.2025.1475323 (PMC11996637; doi:10.3389/fendo.2025.1475323)

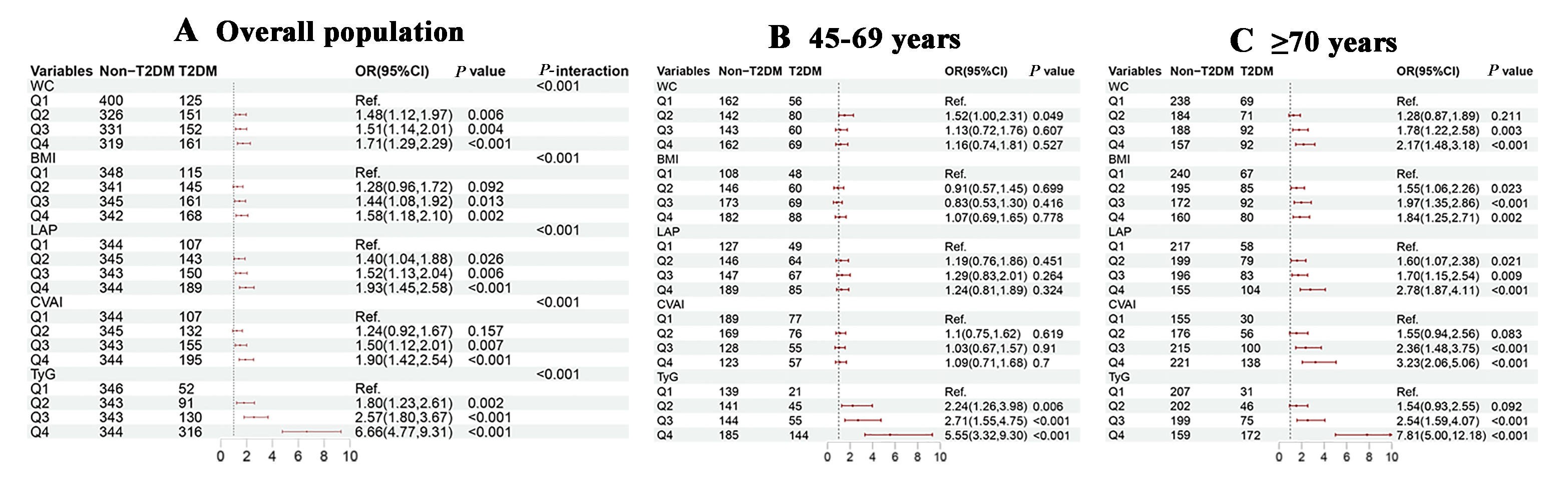

Supplement: Supplementary Figure 1 — Age subgroup analysis in the cross-sectional study. (A) corresponds to Overall population, (B) corresponds to ages 45-69, and (C) corresponds to ages 70 and above. 95%CI, 95% confidence interval; OR, odd ratio; WC waist circumference, BMI body mass index, LAP lipid accumulation product, CVAI Chinese visceral adiposity index, TyG triglyceride-glucose. Adjustment for sex, age, systolic blood pressure, low-density lipoprotein cholesterol, exercise, smoking and alcohol drinking. [file Image1.jpeg]

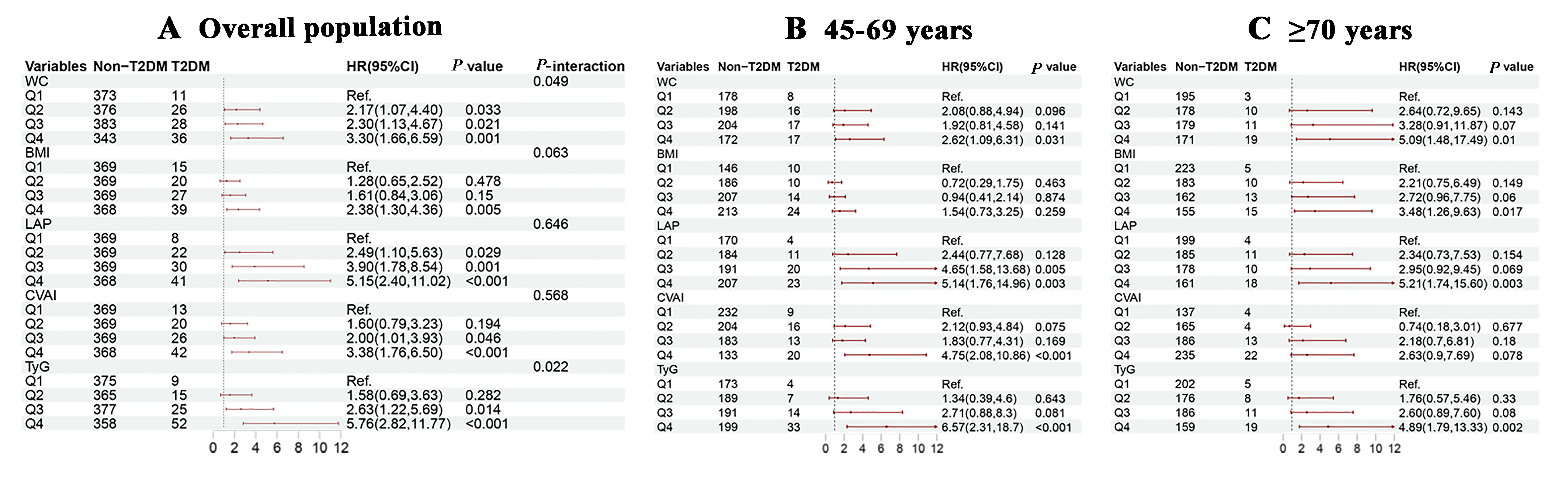

Supplement: Supplementary Figure 2 — Age subgroup analysis in the cohort study. (A) corresponds to Overall population, (B) corresponds to ages 45-69, and (C) corresponds to ages 70 and above. 95%CI, 95% confidence interval; HR, hazard ratio; WC waist circumference, BMI body mass index, LAP lipid accumulation product, CVAI Chinese visceral adiposity index, TyG triglyceride-glucose. Adjustment for sex, age, systolic blood pressure, low-density lipoprotein cholesterol, exercise, smoking and alcohol drinking. [file Image2.jpeg]

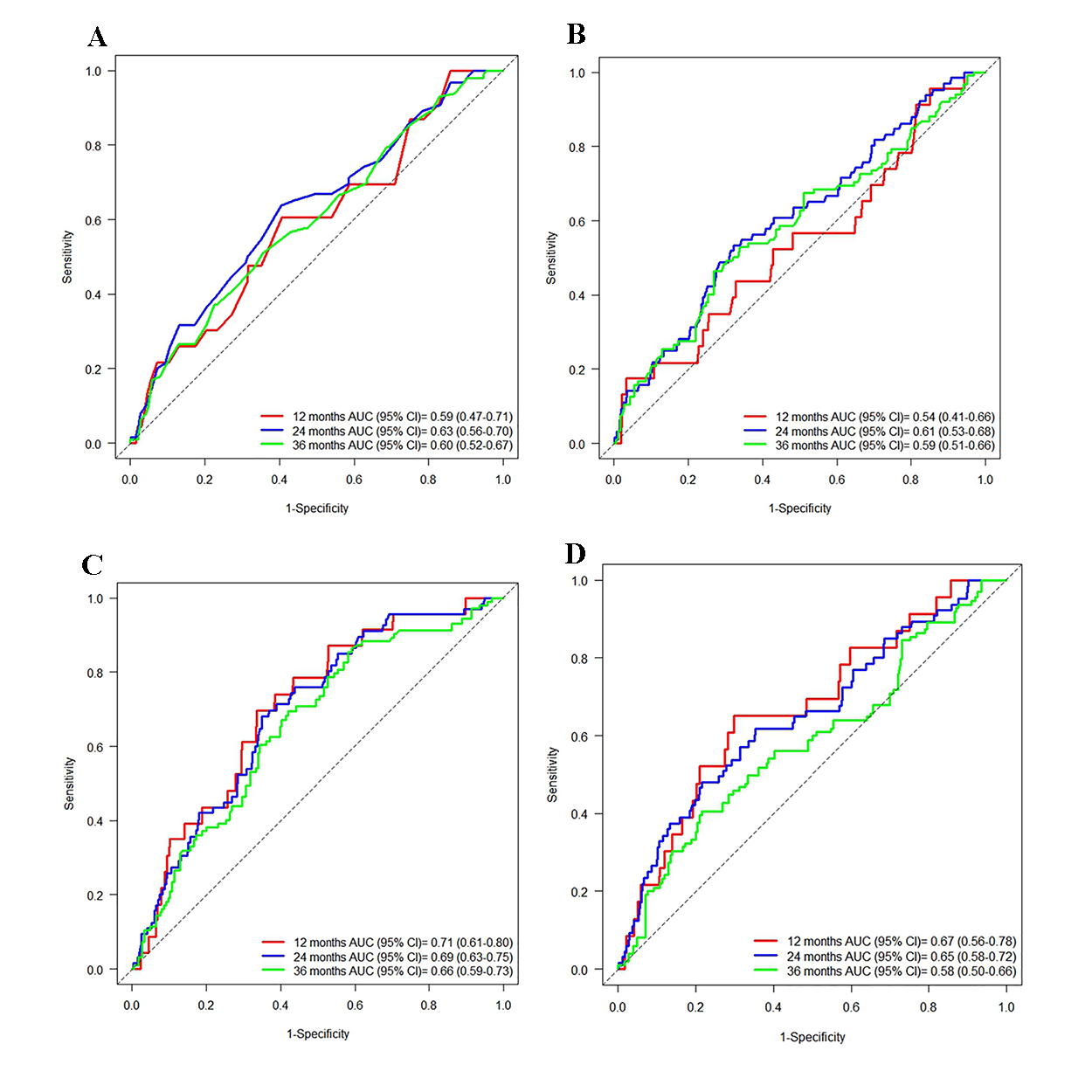

Supplement: Supplementary Figure 3 — Time ROC curves of each index at different time points in the cohort study. (A–D) represent waist circumference (WC), body mass index (BMI), lipid accumulation product (LAP), and Chinese visceral adiposity index (CVAI), respectively. [file Image3.jpeg]
